# A Multi-Scale Approach to Modeling *E. coli* Chemotaxis

**DOI:** 10.3390/e22101101

**Published:** 2020-09-29

**Authors:** Eran Agmon, Ryan K. Spangler

**Affiliations:** Department of Bioengineering, Stanford University, Stanford, CA 94305, USA; spanglry@stanford.edu

**Keywords:** Escherichia coli, chemotaxis, computational systems biology, multi-scale simulation, model integration

## Abstract

The degree to which we can understand the multi-scale organization of cellular life is tied to how well our models can represent this organization and the processes that drive its evolution. This paper uses Vivarium—an engine for composing heterogeneous computational biology models into integrated, multi-scale simulations. Vivarium’s approach is demonstrated by combining several sub-models of biophysical processes into a model of chemotactic *E. coli* that exchange molecules with their environment, express the genes required for chemotaxis, swim, grow, and divide. This model is developed incrementally, highlighting cross-compartment mechanisms that link *E. coli* to its environment, with models for: (1) metabolism and transport, with transport moving nutrients across the membrane boundary and metabolism converting them to useful metabolites, (2) transcription, translation, complexation, and degradation, with stochastic mechanisms that read real gene sequence data and consume base pairs and ATP to make proteins and complexes, and (3) the activity of flagella and chemoreceptors, which together support navigation in the environment.

## 1. Introduction

**Figure 1:**
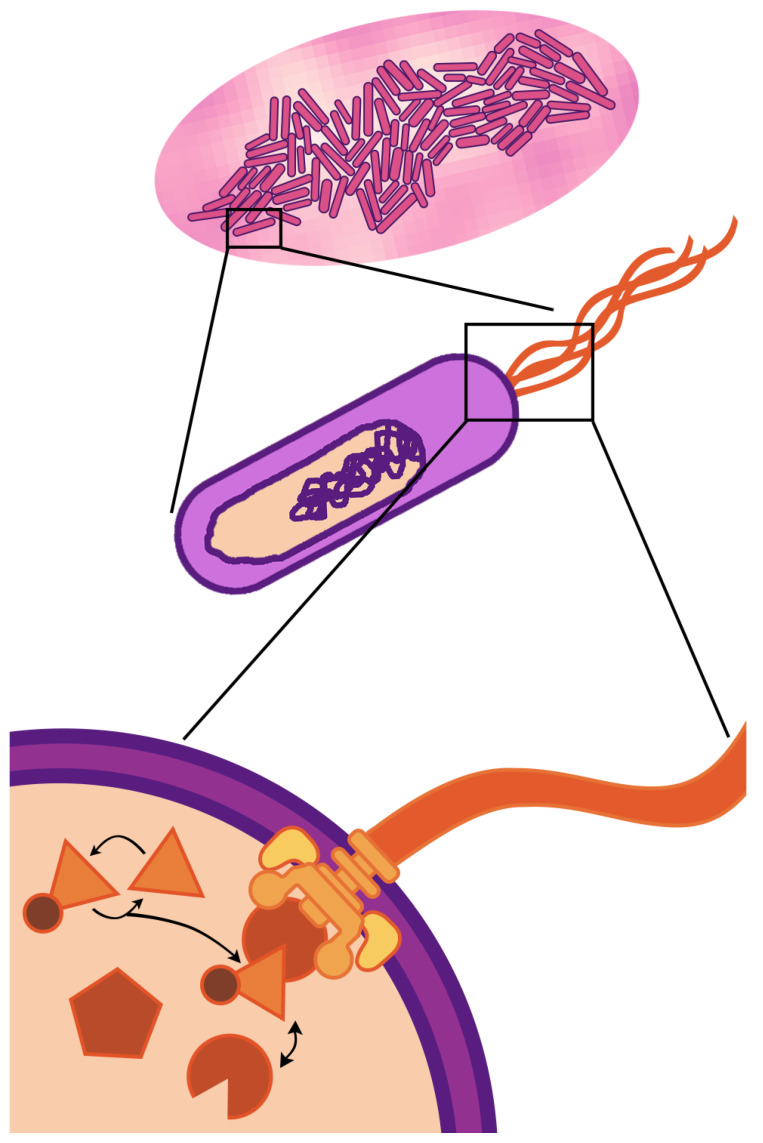
Three scales of *E. coli*. Most fundamentally, *E. coli* are cells (center)—compartments of active molecular processes. Molecules, such as flagella (bottom), have their own structures and dynamics, which, through complex orchestration, support the continued emergence of the cell within which they exist. *E. coli* are also components of a larger system—a colony with a micro-environment (top), which supports and is supported by the cells’ collective function.

The fundamental unit of life—the cell—is a compartment of active molecular processes that operate together as an integrated whole. When we look up close, we see dynamics governed by fluxes of matter and energy that drive molecular reactions, reconfigurations, and transport. When we take a step back, we see the cell is embedded in, and interacts with, an environment filled with other cells, chemical gradients, and physical barriers. Cellular life is fundamentally multi-scale—the physiology and behavior observed at the cellular scale (of micrometer length and second/minute duration) are driven by events occurring at spatio-temporal scales that are both greater and smaller. This paper focuses on multi-scaling of bacterial cells (Figure 1)—the multi-scale nature of life is even more striking in eukaryotic cells, which have internal compartments and can themselves be components of the tissues and organs of multi-cellular organisms.

To confront the complexity of cellular biology, we can encode molecular mechanisms into integrated computational models and simulate them to observe how the hypothesized mechanisms operate as a whole. Where the simulation output matches experimental observations, there might be truth to the proposed mechanisms; where the output diverges, we know something is wrong. Cellular phenotypes emerge through diverse mechanisms operating both internally and externally to the cell—to capture this emergence in realistic detail requires a multi-scale modeling approach that decomposes cellular processes into independent sub-systems, defines them with the most appropriate computational representations, and integrates them into unified and executable models.

*E. coli* is an ideal target for multi-scale cell modeling. These minimal organisms—rod-shaped bacteria about 2 microns long—have undergone more scientific scrutiny than any other organism, being the model organism used to discover the genetic code, DNA replication, and genetic regulation, and they are now a cornerstone of modern synthetic biology. This is why, as early as 1973, Francis Crick and Sydney Brenner talked about coordinating a world-wide effort toward “the complete solution of *E coli*” [1]—an effort that has continued in different forms to the present day [2,3,4,5,6,7,8,9]. *E. coli* has also been a model organism for the molecular biology of behavior, with every aspect of its chemotaxis—*E. coli’s* primary mode of navigation—as the focus of intense scientific investigation [10]. Analysis reveals the remarkable precision and sensitivity of their chemoreceptors [11,12], the maximization of information transmission [13], and trade-offs between accuracy and speed of adaptation [14]. However, in addition to being an informational process, chemotaxis is also a material and energetic process with molecules that need to be continually synthesized and costs that need to be balanced for the chemotaxis phenotype to be preserved [15]—this is the perspective taken in this paper.

*E. coli* chemotaxis has, for the most part, been studied in isolated subsystems, with mechanisms proposed for practically every active molecular process. There are models published on the activity of chemoreceptors [16], signal transduction pathways to the flagella [17,18], and the resulting motion [19], as well as some that have begun to integrate these [20,21]. In the wild, *E. coli* chemotaxis is a highly cross-modal phenotype with many integrated subsystems that coordinate their activities. As a consequence, *E. coli* can achieve a diverse range of chemotactic behavior as it moves between different physiological states and environments [22,23]. As a cell moves up a nutrient gradient, it must express the transporters that take up these nutrients, and its metabolism must restructure to utilize these nutrients as they are brought into the cell. If it succeeds, it can grow, divide, and continue to channel fluxes of matter and energy toward some processes and away from others. For a complete understanding of these interactions, their trade-offs, and the resulting phenotypes, our heterogeneous models of these subsystems must too be integrated.

Vivarium is a novel simulation engine that addresses computational biology’s dual challenges of model reuse and multi-scale integration by explicitly separating the interface that connects models from the frameworks that implement them. This contrasts with standardized modeling approaches, such as the Systems Biology Markup Language (SMBL), which support reuse through a standardized specification of models [24]. Existing multi-scale modeling platforms come packaged with modeling frameworks, including MCell for Monte Carlo models [25], ECell/Spatiocyte for lattice-based stochastic reaction–diffusion models [26,27], CompuCell3D for cellular Potts models [28], CellModeller for cellular biophysics with signaling and gene regulation [29], COMETS for dynamic flux balance analysis with spatial diffusion [30], and Biocellion for discrete agent-based models [31]. Simbiotics is an example most similar to Vivarium, with multi-cell physics and an interface that supports added modules [32]. By embracing the abstraction of an interface for models, users of Vivarium can implement any type of model module they want—whether it is a custom module of a specific biophysical system, a configurable module with its own standardized specification, or an off-the-shelf library with an interface added as a wrapper. This sets the groundwork for a more modular and incremental approach to modeling that supports community development and greater resulting complexity.

This paper applies Vivarium’s multi-scale framework to integrate several models of biophysical mechanisms active during *E. coli* chemotaxis. Section 2 introduces the framework with three multi-scale design principles that outline the interface, the simulation engine, and the incremental modeling approach. Section 3 builds a model of a dynamic spatial environment with a physics engine for the interactions between cell bodies and diffusion operating on molecular fields. Section 4 develops the multi-scale model of chemotactic *E. coli* in three subsections with the progressive integration of new modules: (Section 4.1) Metabolism and transport, with transport moving molecules across the membrane boundary and metabolism converting them into useful metabolites; (Section 4.2) transcription, translation, complexation, and degradation, with stochastic mechanisms that read gene sequence data, consume base pairs and ATP from metabolite pools, and synthesize mRNA, protein monomers, and complexes; (Section 4.3) the activity of flagella and chemoreceptors, which together support chemotaxis in the environment through the coordination of adaptive sensory mechanisms and the production of motile forces. Finally, the discussion in Section 5 argues that no cell model is complete, but with a modular and flexible framework, incremental contributions can build upon past results and asymptotically approach a model that is "whole"—the goal of this paper is to demonstrate how such an approach might work.

## 2. Multi-Scale Design Principles

Vivarium introduces the computational abstraction of a modular “process” interface, which allows different models to be assembled within a hierarchy of embedded compartments and then run as integrated, multi-scale simulations. This section describes the framework with three multi-scale design principles: (1) multi-scale representation of a compartment hierarchy with modular processes, (2) multi-scale simulation with multiple timescales and distributed computation, and (3) multi-scale development of models with modules that can be reused and recombined into increasingly complex computational experiments. This paper’s focus is the application of Vivarium to modeling *E. coli* chemotaxis. For further details on the framework itself, we provide Supplementary Materials along with online documentation and tutorials.

### 2.1. Multi-Scale Representation

**Figure 2:**
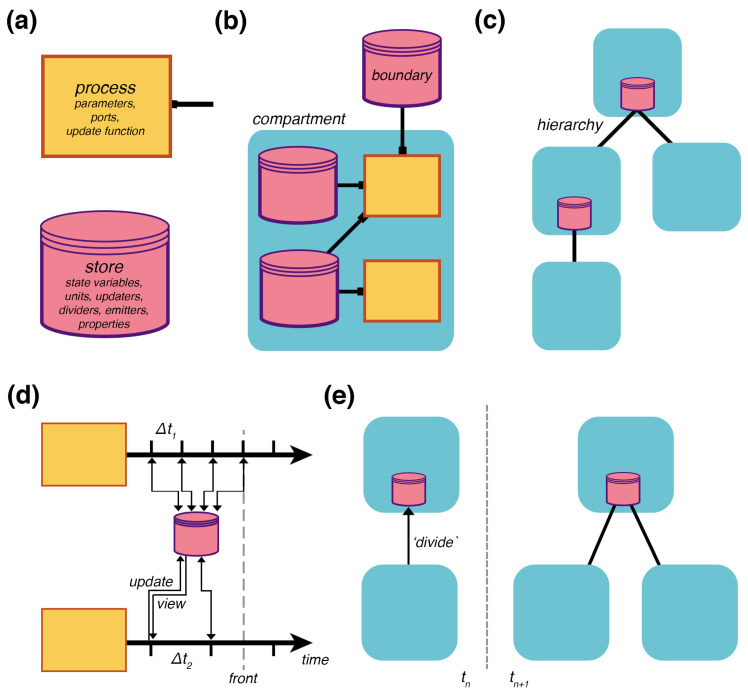
Multi-scale framework. (**a**) Processes and stores are the basic elements of the framework, here represented with flowchart symbols for processes and databases. Processes declare *parameters*, *ports* that connect to stores, and an *update function* that computes how the state variables unfold over time. Stores hold the state variables, map each process’s variable names to their values, and determine how the variables are handled with *units*, *updaters*, *dividers*, *emitters*, *properties* such as mass, and more. (**b**) A *compartment* is a composite of processes created with a bipartite graph called a topology that declares how processes connect to stores through their ports. Boundary stores reach outside of the compartment, allowing it to connect with other compartments above or below. (**c**) A *hierarchy* of embedded compartments is shown as a place graph with the higher compartments containing those below. (**d**) Two coupled processes operating at different time scales, showing their separated updates of a shared store, and an advancing temporal front. At each time-step, a process returns an update that is applied to the store, and the store provides a view of the current state from which the processes resume. (**e**) A topology update shows the addition of a compartment in the time-step after a division update message is sent—other topology updates might include merging, engulfing, deleting, or adding.

The formal structure of a Vivarium model is a *bigraph* (which we also call a compartment hierarchy) of embedded compartments, based on the writings of Robin Milner [33]. A bigraph consists of two structures on the same set of nodes: (1) A *topology* is a bipartite graph of processes that connect to stores (Figure 2b), and (2) a *hierarchy* is a place graph of embedded compartments (Figure 2c). Processes represent the mechanisms, which run internal models to transform the state of their declared variables and send updates back to the hierarchy through their *ports*. *Stores* hold the state of the variables and apply the updates transmitted from the processes (Figure 2a). These can be assembled across different compartments in a hierarchy, and their interactions drive the evolution of the hierarchy’s state as a whole.

Any model that can take a state and transform it for an interval of time can be made into a process by implementing a process interface. The interface is designed to be as simple as possible—it requires the following protocol: (1) A constructor that accepts a list of parameters that configure the model—this overrides default parameter values and allows easy access to parameters by users or by learning algorithms; (2) a ports_schema method that declares the ports and their schema, which determines how updates are handled by the stores—this includes *units* for the variables, *properties* such as molecular weight, *updaters*, which declare how to apply the updates, *dividers*, which declare how each variable is divided amongst daughters, and *emitters*, which declare which variables will be saved for subsequent visualization and analysis; (3) a next_update method, which takes the current state and a time-step, runs the model and returns an update; finally, (4) a composite of multiple processes declares a topology that defines how processes are wired together through shared stores. Composites can also accept parameters, which are passed to their processes’ constructors to override the defaults.

### 2.2. Multi-Scale Simulation

A compartment hierarchy is assembled and executed by the Vivarium engine in a computational object called an *experiment*. A basic simulation cycle has processes view their declared states, run their mechanisms for a set time, and return an update that modifies the states (Figure 2d). A compartment hierarchy’s topology has its own type of constructive dynamics with formation/destruction, merging/division, and encapsulation/excretion of compartments. As with state updates, topology updates are triggered by the processes—Figure 2e shows an update that triggers division. To support large simulations with any number of interconnected processes, the engine can distribute processes onto different threads of execution across an arbitrary number of computers in a cluster; this is done through the passing of a simple flag upon process initialization.

For processes to operate at different timescales, the engine handles updates on a per-process basis. Each process declares its own required time-step, and can update this time-step during runtime. The engine manages the time-steps as it tracks the hierarchy’s current state, the temporal front (Figure 2d). The experiment triggers each process, retrieves, and applies their updates as the front progresses in time. This means some processes can update the shared stores more frequently than other processes. With the current version of the engine, the user needs to make sure the time-steps of the processes are synchronized with each other to avoid numerical issues. Future versions can introduce a specialized adaptor process to handle the processes’ time-steps in a way that automatically ensures coordination.

### 2.3. Multi-Scale Development

Modularity is both a biological and a computational pattern that supports the assembly of large, complex systems by decomposition into smaller, reusable pieces. Modular model design is essential for scientists that wish to confront the complexity of living cells and larger organisms. By separating the process interface from the modeling framework, Vivarium allows users to develop any type of model as a modular process. Categories of possible processes include: (1) Custom modules specific to a given biophysical system. These are the easiest way to adapt models from prior publications, and can be used for their single function. This paper demonstrates these with the chemoreceptor and flagella processes. (2) Wrapper modules built around external modeling libraries—if other platforms are available that can be imported to accomplish the modeling task, they should be reused. This paper demonstrates such processes with pymunk [34] for the multi-body process and COBRA [35,36] for metabolism. (3) Configurable modules with their own standardized specifications—these are the best kinds of processes because configuration data, such as parameters or network structures, can be passed in to make them operate according to different relations. This paper demonstrates configurable processes with the gene expression, enzyme kinetics, and environmental processes.

Further modularity comes from the separation of functions in different software libraries. The Vivarium engine is available as its own library, which is required for implementing the process interface and running a simulation. Individual modeling projects can release their processes, composites, and data in separate libraries. This streamlines model development, allowing users to (1) write their own processes and composites, (2) import libraries from different projects to access their processes, reconfigure them, and combine them to quickly prototype new models, and to (3) make incremental changes (add, remove, swap, reconfigure) and iterate on model designs that build upon previous work. Finally, Vivarium is released under the MIT license, permitting open development and reuse. By open-sourcing the software, future scientists can build upon existing models by adding new processes, making new predictions, and testing against new data.

## 3. Growth and Division in an Environment

*E. coli* can live in a variety of environments of different molecular compositions, which drive patterns of growth, behavior, and evolution. Their small size, of micron scale, exposes them to very different physics from our own. The low ratio of inertial to viscous forces (a low Reynolds number) overwhelms any inertia they might carry [37]. They are under constant thermal agitation from collisions with molecules; in the absence of motile forces, they undergo Brownian motion, which can quickly randomize their location and orientation. This poses a challenge but also opportunities for navigation, as described in a later section on chemotaxis.

**Figure 3:**
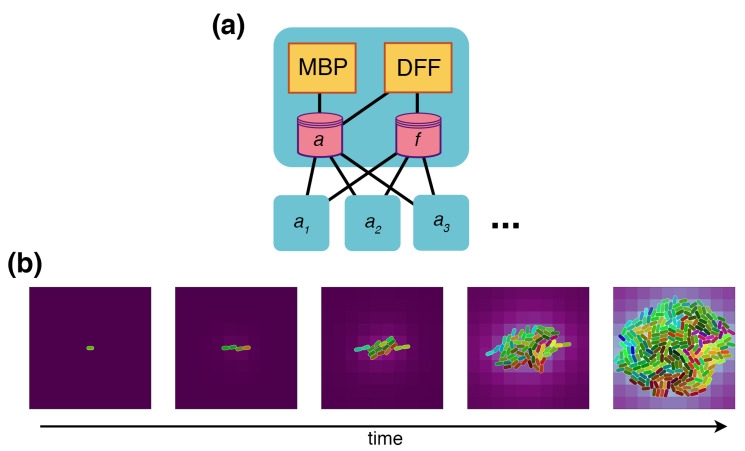
Environments are compartments with sub-compartment “agents”. (**a**) The environment used in this paper is a compartment with processes for multi-body physics (MBP), which simulates agents (in store *a*) as rigid bodies in continuous 2D space, and diffusion (DFF), which diffuses the chemical fields (*f*) in a discretized 2D space and updates the agents’ local environments. Agents ($a_{1}$, $a_{2}$, $a_{3}$, etc.) are sub-compartments of the environment with their own internal processes that update boundary stores *a* and *f*, which hold their shape, volume, mass, motile forces, and the state of their local environments. (**b**) A basic growth/division cell compartment is placed in the environment and simulated. This initial cell is equivalent to one of the agents in panel (**a**). As cells grow and divide, MBP simulates volume exclusion, which pushes neighbors away and grows the colony. The exchange of molecules with the environment generates self-imposed chemical gradients. Cell color represents phylogeny; daughter cells inherit their mothers’ colors with a mutation in color space.

The environment used in this paper is a compartment with two processes—*multi-body physics* and *diffusion*—these operate on the states of agent bodies and molecular fields (Figure 3a). The environmental states exist in a bounded two-dimensional space—agent bodies are capsule-shaped with continuous-valued locations, orientations, volumes, and motile forces; fields are continuous concentration values in a discretized spatial grid. Multi-body physics uses an open-source physics engine [34] to simulate agents as rigid bodies that can move, grow, and collide. Their inertia is highly damped to reproduce the low Reynolds experienced by real *E. coli*, and thermal jitter randomizes their position through forces applied at random to their body. At large colony sizes, this process can become computationally expensive due to the many resulting interactions; the larger simulations described below are flagged for parallelization to improve performance. Diffusion operates on the molecular fields, driving them towards homogeneity. It can support dozens of molecular species, such as ammonium, oxygen, and water, nutrients, such as glucose and the amino acids, and secreted molecules, such as carbon monoxide. The diffusion process updates the state of each agent’s local environment based on its location.

*Growth and division:* It is advised to start minimally when building models, and when satisfied, to expand from there. To test the environment, we add minimal agents with only the capacity to take up nutrients, grow, and divide. Cellular mass is lumped into a single value, and growth is modeled with an exponential function to reproduce *E. coli* exponential growth with a doubling time of about 40 min. Cells are initialized at 1000fg, and division is triggered when the cells reach double that mass; the division condition is configurable, and more complete cell models can replace the mass condition with conditions such as the completion of chromosome segregation or the formation of a septum. The resulting behavior (in Figure 3b) shows how colonies of these minimal agents grow in the environment—as the cells grow, the multi-body process has them push against each other, and the colony increases in area. Some striation can also be observed as a consequence of these interactions. A nutrient in the environment is taken up, which locally depletes the external fields and generates self-imposed gradients around the colony.

## 4. A Multi-Scale Model of *E. coli*

**Figure 4:**
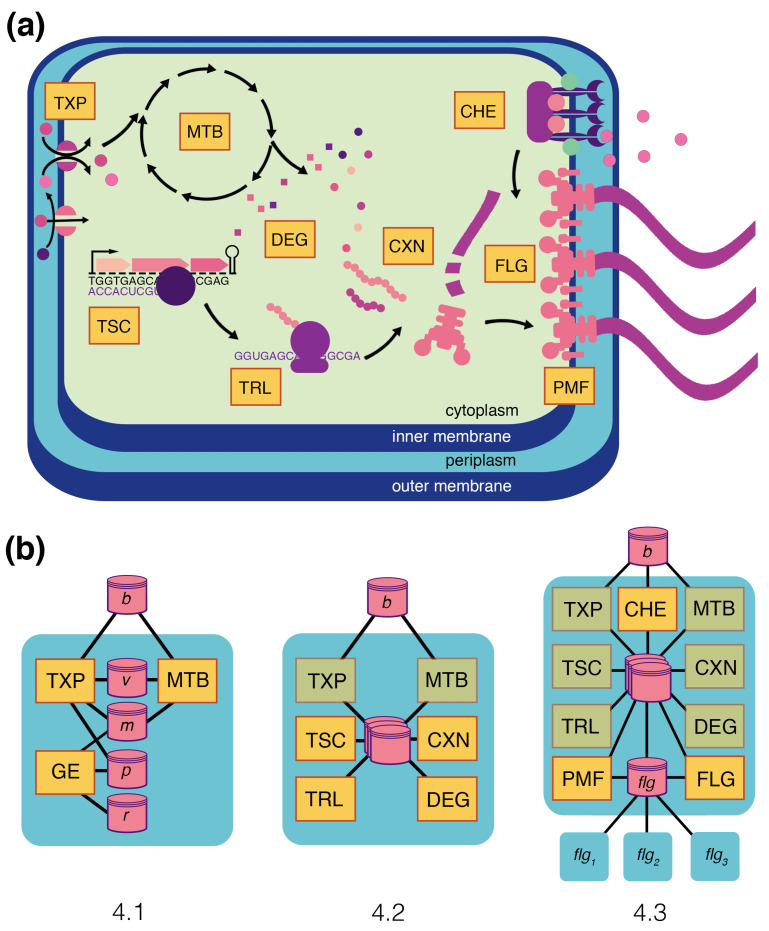
A multi-scale model of *E. coli*. (**a**) A representation of the full model, with its processes as yellow rectangles and stores with the illustrative pink and purple shapes. Processes include metabolism (MTB), transport (TXP), transcription (TSC), translation (TRL), complexation (CXN), degradation (DEG), proton motive force (PMF), flagella activity (FLG), and chemoreceptor activity (CHE). (**b**) The full model is developed incrementally in three subsections; the topologies for each of these models are shown with the corresponding subsection number. Section 4.1 begins with an empty compartment and adds metabolism, transport, and a gene expression process (GE). It has stores for the boundary (*b*), fluxes (*v*), metabolites (*m*), proteins (*p*), and RNA (*r*). Section 4.2 replaces the previous step’s gene expression with stochastic processes of transcription, translation, complexation, and degradation that can take in real sequence data, transcription unit structures, and polymerase binding affinities to synthesize mRNA, proteins, and complexes. These are demonstrated by simulating a flagellar gene expression. Several stores are here shown simplified as overlapping stores. Section 4.3 adds processes for flagella activity, the proton motive force, and chemoreceptor activity, which allow the cell to move within and sense its environment. Flagella are sub-compartments that are added to the cell with the expression of every flagellum complex—each flagellum exerts a motile force that is passed to the environment, and they are split between daughter cells upon division.

This section walks through the composition of a multi-scale model of *E. coli* (Figure 4a). This model includes several processes essential to chemotaxis, including metabolism, transport, transcription, translation, complexation, degradation, the activity of flagella, the proton motive force, and chemoreceptors. The integrated *E. coli* model navigates its environment to find and utilize the resources it needs to survive. This focuses on interactions with changing glucose and lactose in the environment. In reality, *E. coli* can utilize many different carbon, nitrogen, and phosphorous sources, but these drive a diverse range of transcription and translation strategies [38], leading to a range of phenotypes that are not included in this model at the current stage of development.

The model is constructed in incremental steps broken into three sub-sections, as shown in Figure 4b. Each subsection begins with a description of the underlying molecular biology for the given phenomenon, then describes the modeling approaches taken for each process, and ends with an integration of these processes that demonstrates different emergent phenomena. To remain focused on the biology and model integration, only minimal mathematical representations of the processes are included in this main text. Further details on the equations, parameters, and implementation of each described process are provided in Supplementary Materials and in the online documentation.

### 4.1. The Cell’s Supply Line: Transport and Metabolism

Transporters are trans-membrane proteins that move molecules across a membrane, between the cell and its environment. Metabolism takes the imported nutrients, breaks them down, and converts them into the building blocks and energy carriers required for cellular function. Cells optimize their coupled transport/metabolism systems to make the most of their available environmental nutrients. The required transporters are up-regulated, while proteins that compete for membrane real estate and other resources are down-regulated, allowing the cell to maintain steady uptake. The cell’s internal enzyme composition is likewise remodeled to maximize flux from the available internal nutrient pools to the demanded metabolic end-products.

Consider glucose–lactose diauxie as an example of this dynamic process [39,40]. When grown in media containing the two sugars glucose and lactose, a colony of *E. coli* will first consume only the glucose until it is depleted; the colony will then enter a lag phase of reduced growth, which is followed by a second phase of growth from lactose uptake. During the glucose growth phase, the expression of LacY, a lactose transporter, is inhibited, and glucose transporters GalP and PTS are expressed. When glucose is depleted, the cells at first do not have the capacity to import lactose, but *lacY* (along with other *lac* genes) becomes disinhibited and begins to express LacY protein. With sufficient LacY in the membrane, lactose is taken up, the metabolism shifts to lactose metabolism, and growth resumes.

#### 4.1.1. Processes

*Metabolism:* We begin with a dynamic flux balance analysis (dFBA) model of metabolism. Flux balance analysis (FBA) is an optimization-based approach, which takes network reconstructions of biochemical systems and applies linear programming to determine flux distributions in the absence of kinetic parameters. It assumes a steady state, with fluxes across a stoichiometric network of reactions all balanced for each individual molecular species. Constraints are imposed on individual fluxes, setting their upper and lower limits. An objective function is then optimized; the model in this paper optimizes the production of biomass based on known composition of metabolic end-products [41]. FBA is made dynamic by iteratively re-optimizing the objective with updated constraints [42] that can come from changes in environmental nutrient availability, in gene regulation, or in the composition of enzymes that determine flux. These assumptions work well for predicting real flux profiles measured in colonies of cells, one reason being that as colonies grow, those cells that optimize flux are the ones that dominate and are, therefore, experimentally observed.

The metabolism process is configurable and can run any properly formatted metabolic network. The chosen network is a genome-scale metabolic model called *iAF1260b*, which comes from the BiGG model database [36]. The metabolism process can load any BiGG model, including dozens of *E. coli* strains, *Salmonella*, and even some human cells. *iAF1260b* includes 2382 reactions controlled by 1261 genes, which transform 166 metabolites. *iAF1260b* was selected because it includes the relevant transport reactions and generates the four nucleotides and twenty amino acids required for the gene expression processes in Section 4.2. Its objective includes 67 molecules, which are passed into internal metabolite pools that are available for other processes to utilize.

**Figure 5:**
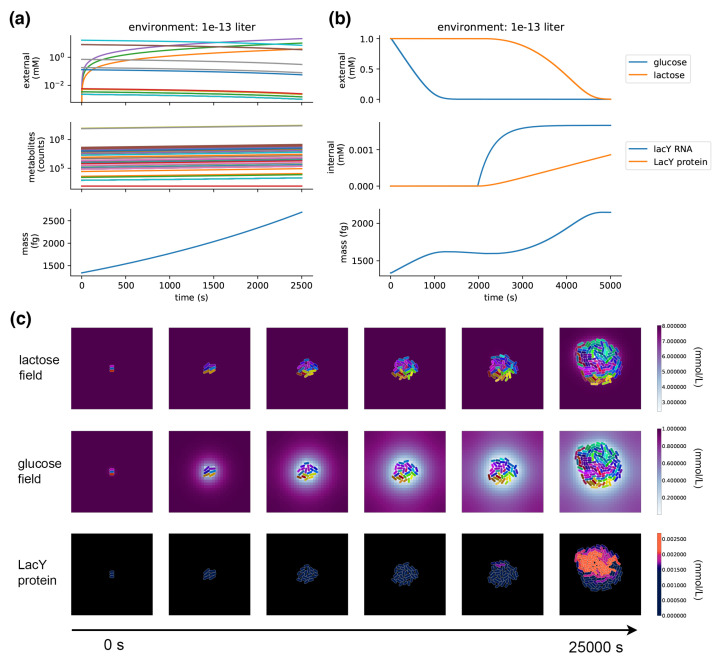
Metabolism, transport, and gene expression. (**a**) Simulation of the metabolism process runs on its own. Environmental (top) and internal molecules (middle) are plotted in log-scale due to the wide range of concentrations. Internal metabolites increase over time, and the environment shows decreasing concentrations for metabolites that are taken up by the cells and increasing concentrations for secreted metabolites (such as carbon monoxide); the total mass of the compartment and environment is conserved. The internal metabolites are multiplied by their molecular weight and summed to get total biomass (bottom), reproducing exponential growth and the doubling time of about 40 min in minimal glucose media. (**b**) A simulated compartment with transport, metabolism, and gene expression processes. The simulation is initialized with low concentrations of glucose and lactose in a small environment (top). At first, glucose is taken in by the cell and the mass increases (bottom). Upon depletion of external glucose, growth slows, and LacY begins to be expressed (middle) and transports lactose into the cell, picking up growth again (bottom). (**c**) An environmental simulation with three initial agents—the top two rows show environmental concentrations of lactose and glucose with agents colored by phylogeny; the bottom row shows a “fluorescent” tag of the LacY protein with brighter colors, indicating more expression. The cells begin by taking up glucose (middle), which starts off close to depleted with a concentration of 1.0 mM and very shallow fields, and thus supports slow initial growth. Some time after glucose is locally depleted, some cells spontaneously switch on LacY expression (bottom); this is no longer inhibited in the absence of glucose, and a feedback cycle brings the more abundant lactose into the cell, fueling increased growth. LacY is already being expressed by the second to last snapshot, though its color is faint because of the relatively much higher expression in the last snapshot. With LacY expressed, those cells begin to take up lactose (top) and grow, overtaking the colony with more widespread LacY expression by the last snapshot.

When a metabolism process is placed in a compartment and run on its own, it takes up metabolites from the environment and grows its internal pools of metabolites, exponentially increasing in mass and reproducing the expected 40 minute doubling time in minimal glucose media (Figure 5a). If metabolites are depleted in the local environment, those exchange fluxes are shut down and FBA finds solutions through other pathways.

*Transport:* If we take the perspective of an individual cell in a colony, the influx of nutrients can no longer be explained by the optimization of biomass production. External molecules are brought into the cells by transporters embedded in their membranes, and it takes an optimized composition of transporters to maximize influx—this composition is under complex mechanistic control involving gene regulation networks and competition with other proteins. Glucose is transported into the cell by two primary transport systems [43,44]: (1) the GalP symporter, which transports glucose with a proton co-factor, and (2) the phosphotransferase system (PTS), a sugar uptake system that uses phosphoenolpyruvate (PEP) as its source of energy. Lactose is transported by LacY, a symporter that transports lactose with a proton as cofactor. Here, each sugar’s transport apparatus is reduced to a single rate function, which determines its total flux across the membrane.

The transport process uses a generalized form of the Michaelis–Menten kinetic rate laws called convenience kinetics [45]. The minimal form of Michaelis–Menten kinetics defines flux rates as a function of substrate concentrations and enzyme concentrations, with parameters for the catalytic rate of a single enzyme, as well as substrates’ affinities. Convenience kinetics is an alternate formulation that can implement all possible Michaelis–Menten kinetic rate laws with any number of substrates, co-factors, and competitor interactions. This was implemented in a configurable process that requires as a parameter a network of the same form as the metabolism process as well as kinetic parameters for each reaction. This configurability to any arbitrary catalytic network and alignment with metabolism makes it highly attractive as a process model. The kinetic parameters for the transport reactions used here were fit to reported growth rates under glucose conditions with exponential growth, lactose conditions with exponential growth, and depleted glucose conditions in the lag phase with low growth.

*Gene expression:* To demonstrate the dynamics of kinetic transport, we need to express transporters and have their state control transport flux. A minimal, stochastic ordinary differential equation (ODE) process for gene expression is used for LacY transporters, which is informed by the schematic of the models and parameters described in [40,46]. This model abstracts over gene copy number, RNA polymerase abundance, strength of gene promoters, and availability of nucleotides. Translation rate abstracts over ribosome availability, strength of ribosome binding to mRNA, availability of tRNAs, and free amino acids. The next subsection replaces this minimal gene expression process with four detailed stochastic processes that account for all of these listed factors.

#### 4.1.2. Integration: Glucose–Lactose Diauxie

Figure 4b shows the topology of the integrated compartment. With FBA for metabolism, kinetic equations for transport, and stochastic ODE for gene expression, this integration is a type of integrative FBA (iFBA) [47]. Metabolism and transport are coupled through a flux store (*v*), with transport setting the dynamic uptake rates, and metabolism using them as flux constraints on the FBA problem. This works because transport reactions operate on the boundary of the metabolic network, and metabolism is limited to use what is supplied by these flux constraints. Transport and gene expression are coupled through a protein store (*p*), which holds the transporter proteins, and metabolite store (*m*), which holds the internal metabolite pools that are built up by metabolism and regulate gene expression. As internal metabolite pools change, this triggers changes to the expression of transporters, which influences the transport rates and, hence, the flux constraints on metabolism.

The integrated model can be placed in the environment and run—it grows and divides, with each daughter cell having its own transport kinetics, metabolism, and gene regulation. This results in, for the first time to our knowledge, genome-scale models of colonies that are implemented on an individual cell basis, with heterogeneous cells distributed in a spatial environment—each carrying out its own FBA instance with dynamic flux constraints. This is already a fairly large model compared to a standard agent-based model.

The model demonstrates the emergence of glucose/lactose diauxic shifts. Figure 5b shows a single compartment simulation of the diauxic shift; Figure 5c shows a population-scale simulation with many cells that grow and divide in a dynamic spatial environment. This experiment starts off nearly depleted of glucose, so the cells start off in a period of arrested growth, which, after a long lag phase, is followed by a switch to lactose metabolism, and growth is resumed. The spatial environment demonstrates how location and crowding can influence the glucose/lactose shift—the shift to lactose metabolism occurs in the center of the colony, where glucose has been depleted. A stochastic burst of LacY expression in one of these centrally located cells triggers the onset of lactose transport and metabolism in the colony—its daughter cells inherit the LacY and quickly overtake their neighbors.

### 4.2. From DNA to Protein: Stochastic Gene Expression

We now replace the ODE-based gene expression process of the previous subsection with more detailed, configurable, and stochastic processes for transcription, translation, degradation, and complexation. These processes operate on gene and protein sequence data, transcription unit structure, binding propensities, and other parameters to express mRNA, proteins, and protein complexes. They use a Gillespie algorithm [48]—a discrete and stochastic method for systems with few reactants, which explicitly simulates each individual reaction. The Gillespie algorithm is used to simulate the binding of polymerases (RNA polymerase or ribosome) onto templates (DNA or mRNA sequences), resulting in mRNA and proteins, as well as complexation reactions that take protein monomers and make protein complexes. For transcription and translation, the algorithm uses a polymerization function to move the polymerases across the DNA or mRNA template at a set elongation rate and pull the required base pairs (nucleotides or amino acids) out of the available metabolite pools; mass is conserved as these base pairs are converted into polymers. Energy requirements for expression are based on ATP hydrolysis for each synthesized or degraded base pair [49]; the ATP is pulled out of a metabolite pool and converted into ADP.

After describing the four processes, they are integrated with the metabolism and transport of the previous subsection and configured to implement a detailed model of flagella expression; in the following section, these flagella will be given a function that enables them to generate forces in the environment.

#### 4.2.1. Processes

*Transcription: E. coli*’s genome is organized in operons, in which multiple genes are expressed as transcription units that are transcribed together as mRNA. The synthesis of a transcription unit starts at a promoter sequence in the chromosome, where RNA polymerase (RNAP) binds to begin transcription, and ends at a terminator, where the RNAP unbinds. The RNAP uses the sequence of nucleotides in an operon as a template to synthesize a matching mRNA transcript, itself a sequence of nucleotides. The energy cost of transcription comes from ATP hydrolysis, with one ATP for each nucleotide elongation.

The transcription process used here can be configured with any chromosome data that include gene sequence data as well as promoters’ and terminators’ locations. The process operates on the state of RNAPs, nucleotides, ATP, and mRNA. The binding of RNAP is implemented by the Gillespie algorithm, with affinities determined by transcription factors for each promoter. Each promoter has a number of binding sites that can be occupied by a number of different transcription factors (or sigma factors, which are treated similarly). The affinity of the RNAP to bind to a given promoter is modulated by the concentration of transcription factors, which either activate or repress that promoter. The higher these thresholds, the more concentration is required to turn it from an empty state to an occupied state, providing a means to gradually introduce components as determined by changing concentrations of the transcription factor. Once bound to a promoter, the RNAP occludes binding of additional RNAPs—it elongates at a rate of 50 base pairs per second and frees up the promoter region when at a distance of 30 base pairs—which allows a transcription unit to be occupied by many RNAPs at different locations.

*Translation:* The translation process operates on the available mRNAs, amino acids, ATP, and free ribosomes; this process does not include tRNA charging. A ribosome binds to an mRNA and uses it as a template for polymerizing a sequence of amino acids at a rate of 22 amino acids per second, with two ATPs used per amino acid for ATP hydrolysis. The same mRNA can be translated by many ribosomes at once, given a minimum occlusion distance of 50 base pairs between them. Upon termination, the protein and ribosome are released into the appropriate stores, and their counts increased. Each transcript, as a potential operon containing the code for many separate proteins, has a series of separate binding affinities for the gene products it encodes. The affinities of these binding reactions are parameters that can be supplied to the model, and can be targeted by parameter selection algorithms.

*Complexation:* Most protein function comes from the activity of complexes, which are composed of many individual protein monomers. These complexes can be constructed in steps, with intermediate complexes combining to form larger complexes. The stoichiometry of a complexation network describes how monomers combine into complexes, and sub-complexes into larger complexes. The complexation process uses the Gillespie algorithm to determine which monomers or complexes combine and when. The propensities for these reactions are parameters that can be supplied for this model; since these values have not been reported, we used the order-of-magnitude estimate of 1000.0 s${}^{-1}$ for each reaction.

*Degradation:* The degradation process uses basic enzyme kinetics (here provided by the enzyme endoRNAse) to reclaim the nucleotide bases from mRNA at a steady rate, with a cost of one ATP per nucleotide degraded. These transcripts are removed from the cytoplasm, and the bases that compose them are returned to the pool of available nucleotides. Proteins are not degraded in the model due to their much longer degradation time in real cells—the loss of protein concentration is due to dilution from growth and division, and this is the loss that has to be countered by ongoing gene expression.

#### 4.2.2. Integration: Flagellar Expression Network

**Figure 6:**
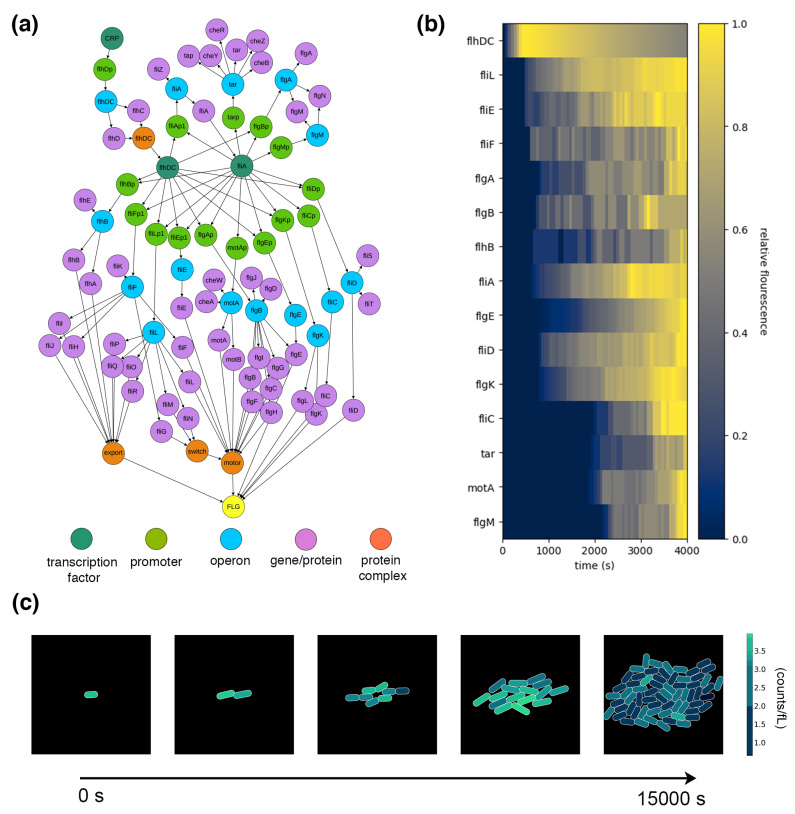
Stochastic expression of flagella. (**a**) The gene regulatory network for flagellar expression with operons, genes, promoters, and transcription factors. Proteins have been compressed into their associated genes in this representation. The final flagellar complex is shown in yellow (FLG). (**b**) Simulated just-in-time expression of flagellar mRNA starting with no initial flagellar expression. This shows transcription factor FlhDC expressed first, and then handing off control to FliA. This is a multi-process behavior that results from the integration of the four described processes. (**c**) “Fluorescent” tags on the final flagellar complex demonstrate heterogeneous protein expression in a small colony, with brightness representing each cell’s concentration of flagella. The simulation begins with a single cell initiated with four flagella and run for 15,000 simulated seconds (~four hours). As cells grow, their concentrations of flagella are diluted, and upon division, the flagella are not evenly divided—the gene expression processes continue to synthesize flagella, so they remain present in the colony.

The gene expression processes can be configured by providing an expression network that includes all of the parameters described above—gene sequences for the operons, promoter and terminator locations, transcription factors for each promoter with threshold values for RNAP binding, and complexation reaction stoichiometry. To demonstrate the integrated function of these processes, we curated a flagellar expression network from the EcoCyc database [50], shown in Figure 6a. Provided with this network, the model exhibits the transcription of the seven flagellar operons, the translation of flagellar proteins, and, ultimately, the complexation of those proteins into intermediate complexes and, finally, the complete flagellum.

Flagella are large protein complexes that require the coordinated expression of many individual monomers. To coordinate the expression of these monomers, *E. coli’s* gene expression machinery has been experimentally demonstrated to perform “just-in-time” expression, with proteins synthesized in roughly the order they are required [51], and proteins that are required together are placed on the same operon and expressed together. This reduces wasted monomer mass and saves the cell physical space and energy.

The key to this expression pattern is a multi-gene feedback loop in the network with two transcription factors—FlhDC and FliA—that get expressed and then take over the regulation of additional required genes. The components’ progressive expression is achieved by an increasing sensitivity to the concentration of FlhDC and an inverse sensitivity to FliA, which is itself regulated by FlhDC. When both are present, FlgM binds FliA and prevents its interaction with RNAP, thus inhibiting FliA-dependent transcription. When the flagellum’s basal body is assembled, FlgM is exported from the cell, initiating the next stage with FliA-dependent transcription. This pattern of transcription activation is configured using a binary sum logic gate blueprint and activation coefficients [52].

Next, polymerases need to be initialized so that they can express the provided flagella genes. There are about 10,000 RNAPs and 20,000 ribosomes in a single *E. coli* [53], which is assumed to be sufficient to express all the required genes for sustained *E. coli* function. The model shown here does not express the full *E. coli* genome, so it is initialized with reduced counts of RNAPs and ribosomes to keep flagella from being over-expressed by an entire cell’s worth of polymerases. Based on an assumption of even distribution based on final protein mass, an average of four flagella were estimated to require 20 ribosomes and 10 RNAPs—these polymerases are themselves not expressed in the model, so they are kept constant for all flagellum-expressing cells and reset upon division.

As shown in Figure 6b, the integrated processes with the included polymerases successfully demonstrate just-in-time expression of flagellar mRNA, with transcripts sequentially increasing in concentration as the simulation unfolds. Figure 6c shows the complete flagellar expression compartment—with transport and metabolism providing the required base pairs and ATP—placed in an environment and simulated. The first cell is initialized with four flagella. The cell’s growth is entirely driven by uptake from the environment, with metabolic end products fueling the gene expression processes. As the cell grows, the concentration of flagella dilutes, but expression counters this reduction by synthesizing new flagella. The stochasticity of expression and variation in nutrient uptake results in a heterogeneous population with different numbers of flagella. Notice that despite there being flagella, the cells remain non-motile—this is because flagella are merely being counted and have not yet been given function.

### 4.3. Motility and Chemotaxis: Flagellar and Chemoreceptor Activity

An *E. coli* flagellum is a reversible rotary motor that is embedded in the cell membrane and generates thrust in the environment [54]. It is powered by a flux of protons across the membrane, driven by an electro-chemical gradient—the proton-motive force (PMF). A flagellum rotates at about 100 Hz either clockwise (CW) or counterclockwise (CCW), as determined by a molecular switch at the flagellum’s base. When all of a cell’s flagella (typically 1–8 in total) are rotating CCW in a normal conformation, they self-assemble into a bundle at one end of the cell and generate forward propulsion in a behavior called a “run”. If any of the flagella rotates CW, it breaks away from the bundle, adding orthogonal thrust that re-orients the cell randomly in a behavior called a “tumble”. As the cell spontaneously switches between run and tumble states, it is driven through a random walk in its environment.

Chemotaxis takes this baseline motility and alters it for directed navigation. The key to navigation is chemoreceptors, which sense changing concentrations in the environment and trigger signal cascades that alter the state of the flagellar motor switch, and thus influence the flagellar rotation direction. This elongates the time of runs when moving up an attractant gradient or down a repellent gradient, generating a biased random walk that brings the cell toward favorable and away from adverse conditions.

There are several classes of *E. coli* chemoreceptors—Tar and Tsr for amino acids and other stimuli, and Trg, Tap, and Aer more specifically for sugars, dipeptides, and redox potential. The cell’s chemoreceptors cluster together at its poles—typically one or two clusters in an entire cell. A cluster activates and deactivates together as a single entity; this amplifies detected signals and increases sensitivity [55]. Chemoreceptors adapt to their environments through the slower negative feedback of methylation by CheR and demethylation by CheB. Adaptive methylation makes the chemoreceptors responsive to relative concentrations over time; this ensures that the chemoreceptors remain sensitive to the direction of increasing concentrations rather than absolute concentrations.

Signal transduction between chemoreceptors and flagellar motors is mediated by a well-known protein interaction network [56]. CheA is bound to the chemoreceptors; it autophosphorylates with chemoreceptor activity and is dephosphorylated by CheZ. When activated, CheA can phosphorylate CheY to make CheY-P, which is, in turn, dephosphorylated by CheZ. If it remains phosphorylated, CheY-P diffuses in the cytoplasm to bind the motor protein FliM at the base of a flagellum. When FliM is bound, it promotes CW rotation.

This subsection takes the flagella expressed in the previous subsection and adds them to the cell as individual sub-compartments with rotational states. Their combined thrust is applied to the agent bodies in the environment, introducing motility. A proton-motive force is integrated to drive their activity, and a chemoreceptor cluster is integrated to sense the environment, trigger signals that control the flagellar rotational states, and generate directed chemotaxis up an attractant gradient.

#### 4.3.1. Processes

*Flagellar activity:* Individual flagella synthesized by the gene expression processes are added to the compartment hierarchy as sub-compartments of a cell. The rotational states of each flagellum are modeled as a stochastic bistable system in which the transition rates between CCW and CW are functions of the phosphorylated protein CheY (CheY-P) [57]—the result of the signal transduction network described below. The aggregate behavior of flagella, which determines the cell’s overall motile state, is adapted from a veto model in which a single flagellum in the CW state puts the cell in a “tumble” [58].

**Figure 7:**
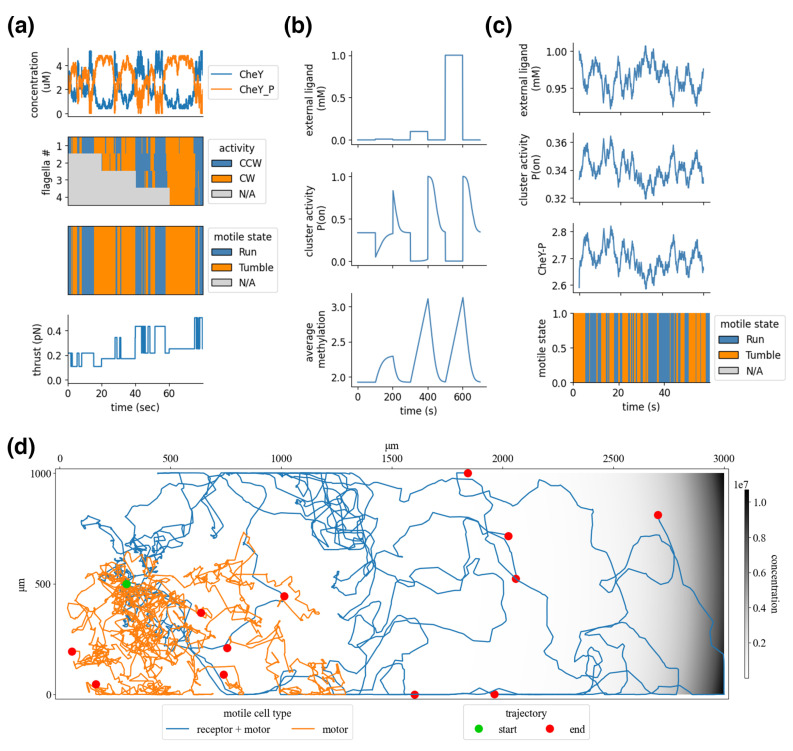
Flagella, chemoreceptors, and chemotaxis. (**a**) The flagellar process is simulated, showing dynamics of CheY phosphorylation, the individual flagellar rotational states, the cells’ motile state, and the generated thrust. The simulation starts with one flagellum, and three more are added during runtime; this alters the thrust as a logarithmic function of the number of flagella. (**b**) The chemoreceptor process, simulated with a pulsing input of the external ligand. When exposed to step functions of different magnitudes, the chemoreceptors adapt through methylation, bringing the chemoreceptor activity back down to a baseline. Pulses of larger magnitude elongate the adaptation time, yet return to the same final steady state. (**c**) The integrated compartment with chemoreceptor and flagellar processes shows the external ligand driving the chemoreceptor cluster activity and CheY phosophorylation, which drive the flagella and overall motile state. (**d**) Twelve cell models are placed at the same starting point in a spatial environment with an exponential MeAsp gradient and are run for eight simulated minutes. Half of the cells have both flagella and chemoreceptors (*motor+receptor*), and the other half only have flagella and defunct chemoreceptors (*motor*). *Motor+receptor* agents show how chemoreceptors bias movement up the gradient, while *motor* agents perform random walks from their starting locations.

Figure 7a shows the “run” and “tumble” states, which generate thrust and torque as a linear function of the PMF and a logarithmic function of individual flagellar rotational states—logarithmic because, as flagella are combined in a bundle, their individual contributions to propulsion are reduced [59]. With a baseline PMF of 140 mV [10] and an expected thrust of approximately 0.5 pN for a swimming bacterium with four flagella [60], the estimated thrust of a single flagellum is here set at 0.31 pN. A “run” applies the combined thrust directly to the back of the cell body, and “tumble” applies the thrust at a random angle. This thrust updates the *agents’* boundary store (Figure 3) and is applied to the agent bodies by the multi-body physics process in the environment—this is described under integration.

The flagellar process also includes the signal transduction model, which calculates the relative steady-state concentration of CheY-P from the rate of CheA autophosphorylation and chemoreceptor activity according to [11,61]. The concentration of CheY-P determines free energy barriers and switching probabilities (from CW to CCW and CCW to CW) between individual flagellar rotational states [57].

*Proton-motive force:* Flagellar motors are driven by an electrochemical gradient of protons across the cell’s inner membrane—the PMF. For every revolution of a flagellum, 1200 protons are moved across the membrane [10]. In a real cell, the PMF is restored across the inner membrane with fast-acting proton pumps; in this model, proton flux is assumed to be instantly restored. The work potential of proton gradients is defined by the sum of the membrane potential and pH difference. The Goldman equation is used for membrane potential and a constant pH difference (which could be added with the Nernst potential). This current model includes cytoplasmic and periplasmic concentrations of ions of potassium, sodium, and chlorine provided by the BioNumbers database [53], giving an initial PMF of –134 mV.

*Chemoreceptors:* The chemoreceptor process implements a model of Tar and Tsr clusters—the amino acid chemoreceptors, which are the most studied chemoreceptors because they can be activated by the non-metabolizable attractant MeAsp and are, therefore, decoupled from metabolism. To get more realistic glucose-specific chemotaxis would require an additional model of Trg in the cluster.

The activity of a chemoreceptor cluster is modeled using a Monod–Wyman–Changeux (MWC) model from [16] and is shown in Figure 7b. The cluster is composed of Tar and Tsr chemoreceptors in a ratio of 1:2. Each chemoreceptor homodimer is modeled as a two-state system (*on* or *off*), with energy values based on ligand concentration and methylation levels. This results in four energy levels: *on* without ligand, *on* with ligand, *off* without ligand, and *off* with ligand. Sensory adaptation comes from the methylation of chemoreceptors, which alters the free-energy offset and transition rate to favor the *on* state; attractant ligand binding favors the *off* state. Adaptation can be seen in Figure 7b, with methylation rising following an increase in external ligand concentrations, which brings the chemoreceptor cluster back to a baseline activity. This methylation dynamic makes the cell responsive to changes in external ligand concentrations, rather than absolute concentrations.

#### 4.3.2. Integration: Chemotaxis

Figure 7c demonstrates the integration of the chemoreceptor and flagella via the signal transduction network, of which CheY-P is shown. Sensori-motor coordination emerges from this integration; as the external ligand concentration increases, the cluster activity drops, which reduces the average Che-P concentrations and biases the flagellar state toward increased CW rotation, and the cell’s motile state favors a run—the inverse is true for reducing ligand concentrations. When the ligand concentration is held relatively constant, the chemoreceptors adapt, and the cell returns to baseline motility. A related study showed that *E. coli* are well suited to navigate up exponential gradients [21].

The resulting thrust and torque are saved in a boundary store and are read by the environment’s multi-body physics process. These motile forces are applied directly to the agents’ bodies along their outer boundary, which pushes them in the environment. A “run” applies the force at a normal angle to the agent’s back end, and a “tumble” applies the force at a random angle. This combines with forces from the environment, such as the viscous force, which dampens inertia, and jitter force, which applies random impulses that randomize the cell’s orientation. The resulting behavior, shown in Figure 7d, generates an average velocity of about 14.1 μm/s (compared with experimentally reported 14.2 μm/s [10]), an average of about 70.0 degree rotation between runs (compared with experimentally reported 68 degrees), run durations of about 1.0 s (0.42 s reported), and tumble durations of 0.7 s (0.14 s reported). As a result of the veto model of flagellar activity, more frequent turns occur when there are more flagella, as observed experimentally [59].

An insight coming from this integration is that the activities of chemoreceptors, flagella, and the environment are more closely coupled than many internal processes need to be to each other. The timescale for chemoreceptors and flagella to achieve successful chemotaxis is a maximum of 0.01 s—longer timescales make the cell miss important gradient information. Additionally, this required the timescale of the environmental processes to also be set at 0.01 s so that they could apply the generated forces and update the cells’ external ligand concentration at a rate that maintains the cell–environment coupling. This can be compared to internal coupling between metabolism and gene expression, which were safely set at 1.0 s.

The simulation time required to approximate growth is in the timescale of hours—six orders of magnitude higher than the required timescale for chemotaxis. The chemoreceptors and flagellar processes could have achieved this, but in order to have the transport/metabolism system respond to environmental changes, they too would need to be brought to a comparable timescale—at a huge computational cost. Furthermore, the spatial scales of the environment required for simulations of growth are enormous relative to the cellular scale because *E. coli* can move at a velocity 10 body lengths per second. Because of this, significant additional work will be required to analyze the consequences of the described model.

## 5. Discussion

This paper took us through a quick tour of the multi-scale development of a chemotactic *E. coli* cell model with a rich internal state and dynamics, which can grow into colonies of heterogeneous cells and move around within a dynamic, spatial environment. The scope of this model is large, and we only scratched the surface of the phenomena implied by the full set of integrated processes. Metabolism, transport, gene expression, flagellar activity, and chemoreception have many reported cross-dependencies that could be more carefully evaluated with a model such as the one described here. Some interesting examples include: (1) Metabolism and chemoreception are linked by the Aer chemoreceptor, which detects redox changes and is thought to respond to the metabolic state of the cell rather than any particular ligand [62]. (2) Transport and chemoreception are linked by CheA, a kinase that transduces signal in the chemotaxis pathway and also interacts with the PTS transport system—this allows glucose to be sensed both by chemoreceptor and by transport systems [63,64]. (3) Stochastic expression of chemotactic proteins leads to individual differences in the chemotactic pathway and flagellum assembly, which generates a wide range of possible motile behaviors [23]. More broadly, the chemotaxis phenotype generated by interactions within a cell intimately links that cell to its environment—a cell’s movement changes its external conditions, which drives the state of its signal systems, its metabolism, and, ultimately, its gene expression and evolution. At the population level, chemotactic cells can exhibit collective swarming behavior, which has been shown to actually remodel their individual expression rates to increase run duration [65]. The opportunities for multi-scale modeling to elucidate these and related interactions are truly boundless.

This multi-scale model is the first application of the interface provided by Vivarium. Building a complete whole-cell model will require a larger project that harnesses the collective effort of many scientists. Each individual cell type has unique mechanisms that have to be curated, with parameters that need to be reconciled, and with distinct phenotypes that have to be validated. For such projects to reach their full potential, we need to refine our modeling frameworks and collaborative coding practices. Vivarium provides an interface for these efforts—a plug-in system that simplifies the integration of alternate process models into complex hybrid, multi-scale simulations. This will lower the barrier to contribution so that new users can encode new processes and plug them into existing models. With good computational design and the assistance of learning algorithms, such a framework will support incremental progress, which, through collaborative effort, can build up an increasingly accurate representation of cells and will one day make them “whole”.

## Supplementary Materials

Vivarium is a collection of open-source python libraries for the composition and execution of integrated multi-scale simulations. The software is freely available at the *vivarium-collective* github page: https://github.com/vivarium-collective. Online documentation provides installation and setup instructions, tutorials, and other informations about the Vivarium framework: https://vivarium-core.readthedocs.io. All of the experiments described in this paper can be accessed in the *vivarium-chemotaxis* repository: https://github.com/vivarium-chemotaxis. Further information about the processes, composites, experiments, and where to find them are provided in this paper’s supplementary materials, https://www.mdpi.com/1099-4300/22/10/1101/s1, which are also linked to in the *vivarium-chemotaxis* repository.
