# Supplementary material for: A Multi-Scale Approach to Modeling E. coli Chemotaxis
Source: Entropy (Basel). 2020 Sep 29;22(10):1101. doi: 10.3390/e22101101 (PMC7597207; doi:10.3390/e22101101)
Supplement: Supplementary file 1 [file entropy-22-01101-s001.pdf]

.

Supplementary Materials for A Multi-Scale Approach to Modeling *E. coli* Chemotaxis

Eran Agmon[1] & Ryan K. Spangler[1]

[1] Department of Bioengineering, Stanford University, Stanford, CA 94305, USA
Correspondence: eagmon@stanford.edu

September 28, 2020

Contents

1 Introduction **2**

2 Processes **2**
2.1 Multi-Body Physics 2
2.2 Diffusion 3
2.3 Metabolism 3
2.4 Convenience Kinetics 4
2.4.1 Configuration Data: Glucose–Lactose Transport 4
2.5 ODE Expression 5
2.5.1 Configuration Data: LacY Expression 6
2.6 Template-Based Stochastic Gene Expression 6
2.6.1 Configuration Data: Flagella Chromosome 7
2.7 Flagella Motor 8
2.8 Membrane Potential 9
2.9 Chemoreceptor Cluster 10

3 Composites **11**

4 Experiments **13**

References **13**

1 Introduction

This supplement provides additional details about all of the process, composites, and experiments used in this paper. To support multi-scale development with many processes developed and combined by large communities of scientists, Vivarium applies principles of modularity to its software design by breaking up functionality into a collection of Python libraries. The library `vivarium-core` is the simulation engine, which provides the interface, combines processes into composites, and executes them in experiments. The core engine can be found at the following url: `https://github.com/vivarium-collective/vivarium-core`. Instructions on installation and setup can be found at the online documentation: `https://vivarium-core.readthedocs.io/en/latest/`.

Process libraries are used to develop specific Vivarium projects with processes and composites that share the Vivarium interface. When released as a Python package, these libraries can be imported into other projects, re-configured, combined with other processes, and simulated in large experiments.

Two process libraries were used to develop the models described in this paper:

- `vivarium-chemotaxis` includes chemotaxis-specific processes built for this paper and all of the paper's experiments:
  `https://github.com/vivarium-collective/vivarium-chemotaxis`.

- `vivarium-cell` provides several configurable process, which can be passed parameters and combined with other processes: `https://github.com/vivarium-collective/vivarium-cell`.

2 Processes

This section provides an overview of each process used in the paper in the order they were introduced. This includes their mathematical representation, the Vivarium library they were developed in, their module path within the library, and configuration data (such as parameters) used in this paper. Config functions are special functions used to retrieve configuration data; they are typically placed within the directory of their associated process/composite. If config functions are listed as 'default', that means there is no separate function available at present and either the default parameters are used or parameters are passed in directly.

There are several additional 'helper' processes not listed here, including `derive_globals`, `derive_concentrations`, `derive_counts`, `tree_mass`, `meta_division`, `divide_volume`, `timeline`, and `nonspatial_environment`. For the curious readers, these are all available in the Vivarium repositories.

2.1 Multi-Body Physics

`Multibody` uses a wrapper around an open-source physics engine called pymunk [1]. This simulates agents as rigid bodies with a shape, location, orientation, length, width, mass, and motile forces that can be updated from within the agents; an example of these forces is

described under Flagella Motor. The multi-body process handles their collisions and applies their motile forces, external viscous forces, and thermal jitter.

Multi-Body Physics	
name	`Multibody`
module path	`cell.processes.multibody_physics`
repository	`vivarium-cell`
config function	`default`

2.2 Diffusion

`DiffusionField` operates on 2D numpy arrays that represent molecular concentration fields. A diffusion operator spreads these concentrations towards a homogeneous state based on provided diffusion coefficients. Agents have a location, which can be updated by `Multibody`, associated with locations on the fields based on their center points. Agents update the fields with their own updaters, applying uptake and secretion directly to the concentrations fields at their given locations; this drives the fields away from homogeneity. `DiffusionField` updates the agents' received local environmental state within its update function.

Diffusion	
name	`DiffusionField`
module path	`cell.processes.diffusion_field`
repository	`vivarium-cell`
config function	`default`

2.3 Metabolism

`Metabolism` uses a wrapper around COBRA [2]—an established library for flux balance analysis (FBA). It can load large genome-scale models from the BiGG model database [6], which includes over 100 metabolic models of different species.

FBA uses linear optimization to determine fluxes in the absence of kinetic parameters. It accomplishes this by using steady-state assumptions to declare $Sv = 0$ with a distribution of fluxes v across a stoichiometric network depicted by matrix S. Constraints are imposed on v, setting their upper and lower bounds. Then, an objective function is optimized—a typical objective function is the maximization of biomass based on known relative composition of required metabolites.

`Metabolism` augments the COBRA solver to make a type of dynamic FBA (dFBA) [12], which iterates on the solver in a piece-wise manner with updated constraints. Constraints come from external nutrient availability, regulation, and flux constraints coming through the process's `flux_bounds` port. This `flux_bounds` port can be used by other processes (in this paper, `ConvenienceKinetics`) to set constraints on metabolism. Additionally, rather than the standard use of the biomass objective to increase a single variable called *biomass*, `Metabolism` takes the constituent metabolites that make up the biomass objective and applies them to internal pools of metabolites (see Figure 5a). This allows other processes to utilize those metabolites.

The paper configures `Metabolism` with *iAF1260b*—a BiGG model of *E. coli* strain K-12, substrain MG1655. *iAF1260b* includes 2382 reactions, 1261 genes, and 166 metabolites.

Metabolism	
name	`Metabolism`
module path	`cell.processes.metabolism`
repository	`vivarium-cell`
config function	`get_iAF1260b_config`

2.4 Convenience Kinetics

The minimal form of Michaelis–Menten kinetics defines flux rates, v, as a function of substrate concentrations, $[S]$, and enzyme concentrations, $[E]$, with parameters for the catalytic rate of a single enzyme, k_{cat}, and substrates' affinities, K_m:

$$v = \frac{k_{cat}[E][S]}{K_m + [S]}$$

Convenience kinetics [8] is a generalized form of the Michaelis–Menten kinetic rate laws, which supports all possible kinetic relations in a single equation:

$$v = \frac{[E](k_{cat,f} * \prod_i a_i^{\alpha_i} - k_{cat,r} * \prod_j b_j^{\beta_j})}{\prod_i(\sum_{m=0}^{\alpha_i} a_i^m) + \prod_j(\sum_{m=0}^{\beta_j} b_j^m) - 1 + \prod_k(\sum_{m=0}^{\gamma_k} c_k^m)}$$

with α_i as the stoichiometric coefficient of substrate i, β_j as the coefficient of product j, $a_i = \frac{[i]}{K_{m,i}}$ as the normalized concentration of i, $b_j = \frac{[j]}{K_{m,j}}$ as the normalized concentration of j, and $c_k = \frac{[k]}{K_{m,k}}$ as the normalized concentration of competitor k. The advantage this provides for computational models lies in the products, $\prod$, which can be evaluated in loops based on the number of cofactors and competitors in the reaction network.

2.4.1 Configuration Data: Glucose–Lactose Transport

`ConvenienceKinetics` accepts configuration data for `reactions`, which is the stoichiometry of the reactions, and `kinetic_parameters`, which are the k_{cat} and K_M for those reactions. The config function `get_glucose_lactose_transport_config` returns the following data for the glucose exchange reaction, EX_glc__D_e, and the lactose exchange reaction, EX_lcts_e. Individual states are annotated parentheses with (port id, variable name)—this is how the `ConvenienceKinetics` process sees states in its update function. Kinetic parameters that have a state correspond to the K_M for that state.

`reactions`:

- EX_glc__D_e:

    stoichiometry:

(internal, g6p_c): 1.0
(external, glc__D_e): -1.0
(internal, pep_c): -1.0
(internal, pyr_c): 1.0

catalyzed by:

(internal, EIIglc)

- EX_lcts_e:

stoichiometry:

(external, lcts_e): -1.0
(internal, lcts_p): 1.0

catalyzed by:

((internal, LacY)

`kinetic_parameters`:

- EX_glc__D_e:

(internal, EIIglc):

(external, glc__D_e): 1×10^{-1} mM
(internal, pep_c): None
kcat_f: 1×10^{2}/s

- EX_lcts_e:

(internal, LacY):

(external, lcts_e): 1×10^{-1}mM
kcat_f: 1×10^{2}/s

2.5 ODE Expression

`OdeExpression` has equations for mRNA and protein production:

$$dM/dt = k_M - d_M * M + \xi$$

$$dP/dt = k_P * M - d_P * P$$

M is the concentration of mRNA, k_M is the transcription rate, and d_M is the transcript degradation rate. P is the concentration of the protein translated from M, k_P is the translation rate, d_P is the protein degradation rate, and ξ is a transcriptional leak that generates small, spontaneous bursts of mRNA expression.

2.5.1 Configuration Data: LacY Expression

LacY expression was configured with the following parameters:

transcription_rates:

lacy_RNA: 5×10^{-6} mM/s

translation_rates:

LacY: 2×10^{-4} mM/s

degradation_rates:

lacy_RNA: 3×10^{-3} mM/s
LacY: 3×10^{-5} mM/s

regulation:

lacy_RNA:
'$if\ [(external, glc\_\_D\_e)\ >\ 0.05\,mM\ or\ (internal, lcts\_p)\ <\ 0.05\,mM]$'

ODE Expression	
name	`ODE_expression`
module path	`cell.processes.ode_expression`
repository	`vivarium-cell`
config function	`get_lacY_expression_config`

2.6 Template-Based Stochastic Gene Expression

Stochastic gene expression processes include `Transcription`, `Translation`, and `Complexation`. These load in several forms of configuration data, including gene sequences, protein sequences, transcription unit structure, binding affinities, and complexation stoichiometry. The processes simulate expression from DNA to protein complexes with stochastic simulations provided by an open-source Gillespie algorithm solver called arrow (`https://github.com/CovertLab/arrow`), which was independently developed by R.K.S. The processes use arrows to simulate the binding and unbinding of ribosomes onto genes and RNAP onto RNA templates, as well as the complexation of the resulting proteins.

The Gillespie algorithm is a stochastic approach to numerically solve discrete chemical systems with known reaction rates. At each iteration, the algorithm calculates the propensities for each reaction given a rate and the counts of the reactants, then selects one reaction to occur and determines the interval of time for the current reaction.

The propensity a_{total} of any reaction to occur is defined as the following, with M total reactions and a as the reaction activity:

$$a_{total} = \sum_{j=1}^{M} a_j$$

To find τ, the time to the next reaction, $rand_1$ is generated from a uniform distribution between 0 and 1, and the following equation is applied:

$$\tau = \frac{1}{a_{total}} ln \left(\frac{1}{rand_1} \right)$$

The reaction that occurs at time τ is found by generating another random number between 0 and 1, $rand_2$, and identifying the smallest q such that:

$$\sum_{j=1}^{q} a_j > a_{total} \cdot rand_2$$

Once bound onto a template, a polymerase goes over provided curated sequences with a specialized `polymerize` function that reads the sequence at a provided elongation rate and pulls the required based pairs out of the available internal pools—if a base pair is absent, this stalls expression. Energy requirements for expression are based on ATP hydrolysis for each synthesized or degraded base pair [11], which is tracked and updated. The parameter polymerase occlusion (set in the paper to 50 base pairs for ribosomes and RNAP) determines how far down the template a polymerase must have moved before another one can bind to the promoter.

2.6.1 Configuration Data: Flagella Chromosome

`FlagellaChromosome` is a knowledge base that holds many different types of data about the flagellar genes: genome sequence, transcription unit structure, affinities, protein sequence, complexation, and reactions. Once instantiated as object `flagella_chromosome`, these configuration data can be accessed and passed into the expression processes.

`flagella_chromosome.sequence` contains the entirety of the *E. coli* genome's nucleotide sequence, loaded from the FASTA file: Escherichia coli str. K-12 substr. MG1655 genome assembly ASM584v2.

`flagella_chromosome.genes` contains the transcription unit structure of all flagellar genes, which were curated from EcoCyc. [5]. The following list shows all included operons followed by a list of their cluster of genes.

- flhDC: [flhD, flhC]
- fliL: [fliL, fliM, fliN, fliO, fliP, fliQ, fliR]
- fliE: [fliE]
- fliF: [fliF, fliG, fliH, fliI, fliJ, fliK]
- flgA: [flgA, flgM, flgN]
- flgB: [flgB, flgC, flgD, flgE, flgF, flgG, flgH, flgI, flgJ]
- flhB: [flhB, flhA, flhE]
- fliA: [fliA, fliZ]
- flgE: [flgE]

- fliD: [fliD, fliS, fliT]
- flgK: [flgK, flgL]
- fliC: [fliC]
- tar: [tar, tap, cheR, cheB, cheY, cheZ]
- motA: [motA, motB, cheA, cheW]
- flgM: [flgM, flgN]

Each operon is regulated by a promoter, which has several relevant values included in `flagella_chromosome.promoters`, which were primarily taken from EcoCyc [5]. This promoter field includes promoter position on the *E. coli* genome, terminator positions, direction of transcription, and terminator affinities.

`flagella_chromosome.protein_sequences` includes all protein sequences for the flagellar genes.

`flagella_chromosome.promoter_affinities` maps from the promoter's binding state to the binding affinity of RNA polymerase. The binding affinities for flagellar transcription are based on a binary SUM gate and activation coefficients of transcription factors FlhDC and FliA [4].

Template-Based Stochastic Gene Expression	
names	`Transcription`<br>`Translation`<br>`Complexation`
module path	`cell.processes.transcription`<br>`cell.processes.translation`<br>`cell.processes.complexation`
repository	`vivarium-cell`
config function	`FlagellaChromosome`

2.7 Flagella Motor

The `flagella_motor` process is a combination of several published models. It models individual flagella as sub-compartments with rotational states and controls the number of flagellar sub-compartments based on the counts of flagellar complexes, which can be controlled by other processes, such as Translation. The total motile activity—a "run" or "tumble"—is a function of the total flagellar activity.

The concentration of phosphorylated CheY, [CheY_P], is found with a steady-state solution of a differential equation from [7; 13]. The equation includes the activity of the chemoreceptor cluster P_{on} from `ReceptorCluster`, the rates of phosphate transfer from CheA_P to CheY $k_Y = 100\mu M^{-1}s^{-1}$, CheZ's dephosphorylation rate of CheY_P $k_Z = 30.0/[CheZ]s^{-1}$, and rate constant $\gamma = 0.1$:

$$[CheY_P] = \left(\frac{k_Y[CheA_P][CheY_{total}]}{k_Y[CheA_P] + k_Z[CheZ] + \gamma}\right)$$

The rotational states of each flagellum are modeled as a stochastic bistable system [10]. The transition rates between CCW and CW are functions of $[CheY_P]$, and arise from thermal fluctuations that push the flagella over free energy barriers between their conformational states. The following equation finds the free energy barrier, ΔG, with parameters for the free energy barrier from CCW→CW $g_0 = 40k_BT$ and the free energy barrier for CW→CCW $g_1 = 40k_BT$; $K_D = 3.06\mu M$ is the binding constant of $\mathrm{CheY_P}$ to FliM at the base of the flagellar motor:

$$\Delta G = \frac{g_0}{4} - \frac{g_1}{2}\left(\frac{CheY_P}{CheY_P + K_D}\right)$$

The switching rates of individual flagella, $k_{CW\to CCW}$ and $k_{CCW\to CW}$, are given by the following, with ω as the characteristic motor switch time:

$$k_{CW\to CCW} = \omega * e^{\Delta G}$$

$$k_{CCW\to CW} = \omega * e^{-\Delta G}$$

With all of the individual flagella in a given rotational state, the veto model of motor activity determines the overall motile state of the cell [9]. Any flagella rotating CW are enough for a "tumble" state; if all are CCW, then the cell is in a "run". The generated thrust is a logarithmic function of the number of flagella and PMF. A run generates this thrust at an angle of 0; a tumble generates it at a random angle. In this paper, the environmental `Multibody` process reads these thrusts and angles and applies them at the back end of each corresponding agent's body.

Flagella Motor	
name	`FlagellaMotor`
module path	`chemotaxis.processes.flagella_motor`
repository	`vivarium-chemotaxis`
config function	`default`

2.8 Membrane Potential

This process is used to calculate the proton-motive force from internal and external concentrations of ions. The proton-motive force, Δp, is comprised of two terms, $\Delta\Psi$ and ΔpH:

$$\Delta p = \Delta\Psi + \Delta pH$$

The Goldman equation gives membrane potential, $\Delta\Psi$:

$$\Delta\Psi = \frac{RT}{F} ln\left(\frac{\sum_i^n P_{C_i}[C_i]_{out} + \sum_j^m P_{A_j}[A_i]_{in}}{\sum_i^n P_{C_i}[C_i]_{in} + \sum_j^m P_{A_j}[A_i]_{out}}\right)$$

with positively charged cations C, negatively charged anions A, and these ions' permeabilities P. Constant R is the ideal gas constant, T is temperature in Kelvin, and F is Faraday's constant.

The transmembrane pH difference, ΔpH, is given by -2.3 kT/e, with Boltzmann constant k, temperature T, and proton charge e. This paper assumed a constant $\Delta pH = -50$ based on measurements for cells grown at a pH of 7.

Membrane Potential	
name	`MembranePotential`
module path	`chemotaxis.processes.membrane_potential`
repository	`vivarium-chemotaxis`
config function	`default`

2.9 Chemoreceptor Cluster

The model of chemoreceptor clusters comes from [3]. This Monod–Wyman–Changeux model includes receptor homodimers, r, with parameters for Tar ($r = Tar$) and Tsr ($r = Tsr$). Each homodimer can be in either an on or off state, and can be either bound or not bound to ligand L; therefore, each has four possible free-energy values.

A piece-wise linear model calculates free energy offsets $\epsilon_r(m)$ from the receptors' average number of methyl groups $m = 0, ..., 8$. Given the offsets, the following equation determines the free energy $f_r(m)$ of each receptor r with dissociation rates of the ligand from receptor in on/off states, $K_r^{on/off}$:

$$f_r = \epsilon_r(m) + log\left(\frac{1 + [L]/K_r^{off}}{1 + [L]/K_r^{on}}\right)$$

The total free energy is the sum of the individual free-energy difference by their relative counts: $F = n_{Tar} f_{Tar} + n_{Tsr} f_{Tsr}$. This determines the rate of receptor activation, P_{on}:

$$P_{on} = \frac{1}{1 + e^F}$$

Receptor adaption comes from the changing methylation levels m based on the concentration of methylating enzyme CheR and demethylating enzyme CheB, cluster activity P_{on}, methylation and demethylation rates k_{meth} and k_{demeth}, and adaptation rate k_{adapt}:

$$\frac{dm}{dt} = k_{adapt}(k_{meth}(1 - P_{on})[CheR] - k_{demeth} P_{on}[CheB])$$

Parameters from [3] were used: $n_{Tar} = 6$, $n_{Tsr} = 12$, $K_{Tar}^{off} = 0.02$, $K_{Tar}^{on} = 0.5$, $K_{Tsr}^{off} = 100.0$, $K_{Tsr}^{on} = 10e6$, $k_{meth} = 0.0625$, $k_{demeth} = 0.0714$.

Chemoreceptor Cluster	
name	`ReceptorCluster`
module path	`chemotaxis.processes.chemoreceptor_cluster`
repository	`vivarium-chemotaxis`
config function	`default`

3 Composites

The primary focus of the main text was the composition of the prior listed processes into compartments. Since much emphasis has been placed on that methodology, this supplementary section on composites is primarily to provide information on where to find the composite objects in order to simulate them.

The topologies of each composite are accessible online at the provided module path. The following example of a topology for `Lattice` demonstrates the general form—an embedded python dictionary with keys at the top level for each process name, with subdictionaries that connect their ports to declared stores. Each process—in this case, multi-body and diffusion—maps its ports to a store using a path relative to the compartment with a python tuple. If the store is at the same compartment level as the process, the path is just `('store_name',)`; to go up one level, a boundary store can use `('..', 'store_name',)`, or to go down one level, `('path_to', 'store_name')`.

```
lattice_topology = {
    'multibody': {
        'agents': ('agents',)},
    'diffusion': {
        'agents': ('agents',),
        'fields': ('fields',)}}
```

Lattice Environment	
name	`Lattice`
module path	`cell.composites.lattice`
processes	`Multibody`<br>`DiffusionField`
repository	`vivarium-cell`
config function	`make_lattice_config`

Static Lattice	
name	`StaticLattice`
module path	`cell.composites.static_lattice`
processes	`Multibody`<br>`StaticField`
repository	`vivarium-cell`
config function	`default`

Growth Division	
name	`GrowthDivision`
module path	`cell/composites/growth_division.py`
processes	`GrowthProtein`<br>`ConvenienceKinetics`<br>`MinimalExpression`
repository	`vivarium-cell`
config function	`default`

Transport Metabolism Expression	
name	`TransportMetabolismExpression`
module path	`chemotaxis/composites/transport_metabolism.py`
processes	`Metabolism`<br>`ConvenienceKinetics`<br>`ODE_expression`
repository	`vivarium-chemotaxis`
config function	`default`

Flagella Expression Metabolism	
name	`FlagellaExpressionMetabolism`
module path	`chemotaxis/composites/flagella_expression.py`
processes	`Metabolism`<br>`ConvenienceKinetics`<br>`Transcription`<br>`Translation`<br>`Complexation`<br>`Degradation`
repository	`vivarium-chemotaxis`
config function	`default`

Chemotaxis Master	
name	ChemotaxisMaster
module path	chemotaxis/composites/chemotaxis_master.py
processes	Metabolism<br>ConvenienceKinetics<br>Transcription<br>Translation<br>Complexation<br>Degradation<br>FlagellaMotor<br>MembranePotential<br>ReceptorCluster
repository	vivarium-chemotaxis
config function	default

4 Experiments

All experiments described and plotted in this paper are available in the `vivarium-chemotaxis` github repository, in the file: `chemotaxis/experiments/paper_experiments.py`. There is a well-commented function for every figure shown in the paper, with in-line comments that outline how the processes, composites, and experiments are configured.

To run these from the command line within the repository:

```
$ python chemotaxis/experiments/paper_experiments.py [exp_id]
```

`exp_id` corresponds to a figure number from the paper. These figure numbers are listed here, along with the processes/composites they use:

- 3b – `GrowthDivision` and `Lattice`
- 5a – `Metabolism`
- 5b – `TransportMetabolismExpression`
- 5c – `TransportMetabolismExpression` and `Lattice`
- 6a – `FlagellaExpressionMetabolism`
- 6b – `FlagellaExpressionMetabolism`
- 6c – `FlagellaExpressionMetabolism` and `Lattice`
- 7a – `FlagellaMotor`
- 7b – `ReceptorCluster`
- 7c – `ChemotaxisMaster`
- 7d – `ChemotaxisMaster` and `StaticLattice`

References

[1] Blomqvist, V. (2007–2019). Pymunk. http://www.pymunk.org/.

[2] Ebrahim, A., Lerman, J. A., Palsson, B. O., and Hyduke, D. R. (2013). Cobrapy: constraints-based reconstruction and analysis for python. *BMC systems biology*, 7(1):74.

[3] Endres, R. G. and Wingreen, N. S. (2006). Precise adaptation in bacterial chemotaxis through assistance neighborhoods. *Proceedings of the National Academy of Sciences*, 103(35):13040–13044.

[4] Kalir, S. and Alon, U. (2004). Using a quantitative blueprint to reprogram the dynamics of the flagella gene network. *Cell*, 117(6):713–720.

[5] Keseler, I. M., Mackie, A., Santos-Zavaleta, A., Billington, R., Bonavides-Martínez, C., Caspi, R., Fulcher, C., Gama-Castro, S., Kothari, A., Krummenacker, M., et al. (2017). The ecocyc database: reflecting new knowledge about escherichia coli k-12. *Nucleic acids research*, 45(D1):D543–D550.

[6] King, Z. A., Lu, J., Dräger, A., Miller, P., Federowicz, S., Lerman, J. A., Ebrahim, A., Palsson, B. O., and Lewis, N. E. (2016). Bigg models: A platform for integrating, standardizing and sharing genome-scale models. *Nucleic acids research*, 44(D1):D515–D522.

[7] Kollmann, M., Løvdok, L., Bartholomé, K., Timmer, J., and Sourjik, V. (2005). Design principles of a bacterial signalling network. *Nature*, 438(7067):504–507.

[8] Liebermeister, W. and Klipp, E. (2006). Bringing metabolic networks to life: integration of kinetic, metabolic, and proteomic data. *Theoretical Biology and Medical Modelling*, 3(1):42.

[9] Mears, P. J., Koirala, S., Rao, C. V., Golding, I., and Chemla, Y. R. (2014). Escherichia coli swimming is robust against variations in flagellar number. *Elife*, 3:e01916.

[10] Sneddon, M. W., Pontius, W., and Emonet, T. (2012). Stochastic coordination of multiple actuators reduces latency and improves chemotactic response in bacteria. *Proceedings of the National Academy of Sciences*, 109(3):805–810.

[11] Thornburg, Z. R., Melo, M. C., Bianchi, D., Brier, T. A., Crotty, C., Breuer, M., Smith, H. O., Hutchison III, C. A., Glass, J. I., and Luthey-Schulten, Z. (2019). Kinetic modeling of the genetic information processes in a minimal cell. *Frontiers in Molecular Biosciences*, 6.

[12] Varma, A. and Palsson, B. O. (1994). Stoichiometric flux balance models quantitatively predict growth and metabolic by-product secretion in wild-type escherichia coli w3110. *Applied and environmental microbiology*, 60(10):3724–3731.

[13] Vladimirov, N., Løvdok, L., Lebiedz, D., and Sourjik, V. (2008). Dependence of bacterial chemotaxis on gradient shape and adaptation rate. *PLoS Comput Biol*, 4(12):e1000242.
